# Phase Change Materials Application in Battery Thermal Management System: A Review

**DOI:** 10.3390/ma13204622

**Published:** 2020-10-16

**Authors:** Changcheng Liu, Dengji Xu, Jingwen Weng, Shujia Zhou, Wenjuan Li, Yongqing Wan, Shuaijun Jiang, Dechuang Zhou, Jian Wang, Que Huang

**Affiliations:** 1 School of Environment and Safety Engineering, North University of China, Taiyuan 030051, China; ccliu@nuc.edu.cn (C.L.); s1914074@st.nuc.edu.cn (S.Z.); s1914036@st.nuc.edu.cn (W.L.); s1914068@st.nuc.edu.cn (Y.W.); s1914060@st.nuc.edu.cn (S.J.); 2 College of Materials Science and Engineering, North University of China, Taiyuan 030051, China; djxu0407@163.com; 3 State Key Laboratory of Fire Science, University of Science and Technology of China, Hefei 230026, China; wengjw@mail.ustc.edu.cn

**Keywords:** lithium-ion battery, phase change materials, battery thermal management system, thermal storage

## Abstract

The purpose of a battery thermal management system (BTMS) is to maintain the battery safety and efficient use as well as ensure the battery temperature is within the safe operating range. The traditional air-cooling-based BTMS not only needs extra power, but it could also not meet the demand of new lithium-ion battery (LIB) packs with high energy density, while liquid cooling BTMS requires complex devices to ensure the effect. Therefore, phase change materials (PCMs)-based BTMS is becoming the trend. By using PCMs to absorb heat, the temperature of a battery pack could be kept within the normal operating range for a long time without using any external power. PCMs could greatly improve the heat dissipation efficiency of BTMS by combining with fillers such as expanded graphite (EG) and metal foam for their high thermal conductivity or coordinating with fins. In addition, PCMs could also be applied in construction materials, solar thermal recovery, textiles and other fields. Herein, a comprehensive review of the PCMs applied in thermal storage devices, especially in BTMS, is provided. In this work, the literature concerning current issues have been reviewed and summarized, while the key challenges of PCM application have been pointed out. This review may bring new insights to the PCM application.

## 1. Introduction

**Figure 1:**
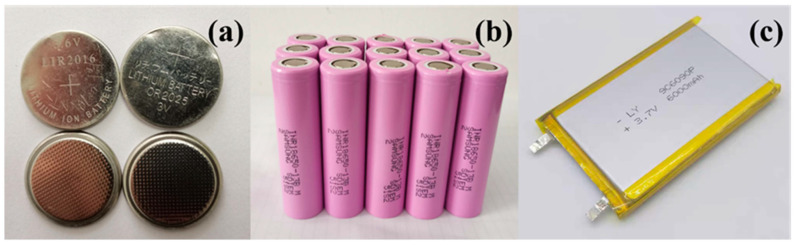
Commonly used lithium-ion batteries (LIBs) with different shapes in daily life. (**a**) Coin cells, (**b**) Cylinder cells, (**c**) Pouch cells.

Nowadays, with the rapid growth of energy demand and the increasing environmental awareness in the society, more and more new energy development and utilization is appreciated by countries all over the world [1,2,3,4,5]. New energy resources such as solar energy, wind energy, tidal energy, geothermal energy, and chemical batteries have developed vigorously in the last few decades, forming an industrial belt of a certain scale. As shown in Figure 1, among all chemical batteries, lithium-ion batteries (LIBs) are widely used in portable electronic products such as smart phones due to the high energy density, long cycling life, high operating power, and environmentally friendly property [6]. In spite of these advantages, there are still some disadvantages that remain. Owing to the high energy density, LIBs are very sensitive to external conditions and factors such as high temperature, overcharge, and short circuit [7,8,9]. In general, the storage temperature range of commonly used LIBs is from 0 to 50 °C. Therefore, the safety problems of LIBs have been increasingly significant with their wide application. Almost every year, there were several safety accidents occurring, which were caused by LIB explosion or combustion induced by a short circuit, overheating, or overcharge. The role of the battery thermal management system (BTMS) is to ensure the operation temperature of the battery pack in a normal safe range. Under overheating conditions, due to the high thermal conductivity performance of phase change materials (PCMs) and the presence of cooling devices such as heat sinks and heat pipes in BTMS, the temperature of a battery pack could quickly return to the normal working range, thus effectively reducing the occurrence of thermal runaway (TR) or fire accidents.

It is significant to understand the mechanisms of battery TR before finding solutions to improve LIBs safety. At present, the TR is mainly caused by the sharp temperature rise inside LIBs induced by abuse or reasons such as short-circuit, overcharge, high-rate charge/discharge and so on, which would eventually lead to the combustion or explosion of the battery. Since most LIBs work mostly in the form of battery packs, each cell could be regarded as a domino, while the TR process of the battery is like dominoes falling. Therefore, under the TR condition (as the Domino Effect), the TR of a single battery might cause the TR of all of the battery groups, resulting in a fire accident. The TR phenomena contain a large amount of heat release, internal gas injection, violent combustion or even explosion of the battery. If TR could not be controlled in time, a majority of fire accidents would subsequently occur. As an example, at 17:00 on 29 February 2020, fire broke out in the equipment room at the northwest corner of the parking lot on the fourth floor in a Costco Supermarket (No. 235 Zhujian Road, Huacao Town, Minhang District, Shanghai, China). Based on the investigation, the fire area on the scene was about 5 m^2^, and there were no casualties. The reason for the fire accident was that the LIBs for the electric tools stored in the machine room broke down and caused the spontaneous combustion, igniting the surrounding combustibles, thus causing a fire. There were many fire accidents caused by TR, as listed and detailed in Table 1.

How to solve the TR during the using process of LIBs has become a key challenge in the entire battery safety industry and gained more and more attention from the researchers. Thus, it is necessary to explore a battery thermal management technology with excellent heat dissipation performance, which could effectively protect the remaining batteries in the pack from damage, even if TR occurs in a single battery. A series of researches on heat dissipation management technology have been carried out. In order to improve the safety of LIBs and avoid the degradation of their performance, it is necessary to design a scientific and reasonable BTMS. Since the factors such as electrochemical performance, battery life and cost directly influence the drivability, life span, costing and economic performance of EVs and hybrid electric vehicles (HEVs), the battery should be working in an optimum temperature range, which is different from various batteries. In addition, the effect of uneven temperature distribution on a battery pack needs to be considered. Nowadays, the manufactures of EVs, HEVs and batteries have realized the issue, and the main solution is adding BTMS. Taking EVs as an example, even though the extra BTMS would decrease the power density of batteries, it could increase the energy utilization efficiency and keep batteries in ideal conditions, then the final cruising range would be extended. Furthermore, BTMS could be helpful to improve the safety of battery packs, drivers and passengers. In addition, before designing BTMS, the influence factors and methods such as cooling method, system structure, weight, and cost need to be considered.

According to different cooling mediums, currently there are five kinds of cooling methods: air cooling, liquid cooling, PCMs cooling, heat pipe cooling [10] and compound cooling. At the same time, according to the condition of whether it is driven by energy, the cooling methods could also be divided into active cooling and passive cooling. Among them, the cooling method at the cost of energy consumption is called active cooling, such as air-conditioning refrigeration and refrigerator cooling, etc. Similarly, the cooling method that does not consume energy at the cost of cooling is named passive cooling, such as natural convection cooling and PCM-based cooling. Compared with the traditional air cooling, liquid cooling, and heat pipe cooling, the PCM cooling method is generally considered to be the most suitable one for BTMS due to its low cost, simple equipment, and high cooling efficiency (specific and detailed reasons are listed in Section 6).

In recent years, PCM-based BTMS has attracted more and more attention from researchers due to its excellent temperature control performance, simple structure, and it is without energy consumption. Hallaj and Selman et al. [11,12] first proposed the idea of PCM application in BTMS. The experimental results showed that at different depths of discharge (DOD), the heating range of the battery cooled with PCM was smaller than that without PCM. However, the commonly used PCM, such as paraffin (PA), had some natural weakness such as low thermal conductivity and easy leakage, which should be modified. Considering the low thermal conductivity of PA, Goli et al. [13] prepared a kind of composite PCM with better thermal conductivity by adding graphene filler. Compared with traditional PA, the thermal conductivity was correspondingly improved by 60 times after adding 1 wt % graphene filler. Wu et al. [14] adopted copper mesh (CM) as a skeleton to protect PA/expanded graphite (EG) composite materials, as a kind of composite PCMs for BTMS. EG with a porous structure could absorb liquid phase PA and prevent the leakage; CM as a module skeleton could further improve the thermal conductivity and strength of the whole module. The experimental results indicated that, comparing PCM without CM, CM/PA/EG had superior temperature uniformity as well as heat dissipation performance. In addition, under special conditions of forced convection, the CM heat sink embedded in the composite PCM not only played a pivotal role in heat dissipation, but also further improved the heat transfer capability by disturbing the airflow. Zhang et al. [15] used kaolin/EG/PA as the PCM in BTMS. By analyzing the influence of different filler proportions on the temperature control performance, the composite PCM consisted of 10 wt % EG, 10 wt % kaolin and 80 wt % PA had the best performance. In the thermal management test, there was no visible leakage after 30 min at a temperature of 60 °C; with a discharge rate of 4C, the highest temperature of a single cell could be effectively controlled below 45 °C, and even under the extreme conditions of higher discharge rate, the temperature difference between each individual cell could also be controlled within 5 °C. Meanwhile, the composite material reduced the temperature of the battery pack by 25.77% at a 4C rate. Thus, once phase change occurred, the PCM could greatly reduce the temperature of the battery pack and even each cell in the pack.

Besides improving the basic properties of PCM, some researchers also paid attention to other influencing factors. The low thermal conductivity of the most pure PCM substance is still a main barrier for further application [16]. Javani et al. [17] studied the influence of PCM thickness on the temperature of PCM-wrapped batteries. The results demonstrated that, when the battery was charged and discharged with different power densities at 21 °C, the temperature distribution became uniform when the thickness of PCM was 3 mm; when the thickness of PCM was 12 mm, the maximum temperature was reduced by 3.04 °C. In the first 7 min, the existence of PCM would be the main reason to alleviate the temperature rise of the battery, and the PCM with larger thickness around the battery could provide a better cooling effect. Ling et al. [18] studied the performance of forced air cooling and PCM-based composite BTMS, and compared the performance between PCM/natural air cooling-based composite BTMS and PCM/forced air cooling-based composite BTMS. The results indicated that the former with low-efficient natural air cooling led to the heat accumulation of PCM under extreme conditions like hyperthermal environments, and it was difficult to export heat to the outside effectively. In the latter experiment, PCM was combined with a forced air convection way, and it was found that the corresponding BTMS could successfully control the maximum temperature of the battery pack below 50 °C at any rate lower than 2C, even if it was 7 °C higher than the ambient temperature, and the introduction of forced convection would not affect the uniform distribution of the temperature provided by PCM. In addition, PCM and forced air convection played different roles in BTMS. PCM controlled the maximum temperature and the temperature difference, while forced air convection cooled PCM. Zhao et al. [19] adopted the method of releasing heat absorbed by PCM through a heat pipe (HP), and carried out detailed design and experimental tests on BTMS coupled with PCM/HP. The results showed that under the same conditions, compared with BTMS based on air and PCM, the PCM/HP coupling could control the maximum temperature below 50 °C for a longer time. By inserting HP into PCM, the temperature difference could be reduced by 28.9%.

In summary, although many researchers focus on PCMs at present, there are insufficient systematic reviews summarizing methods for increasing the thermal conductivity. This work aims to provide a comprehensive summary of some related issues, such as the classification of PCMs, the methods to increase the thermal conductivity, and the application of PCMs in BTMS and some other fields. In this review, the characteristics, classifications and disadvantages of PCM were systematically described, and how to increase the PCM thermal conductivity was summarized, then the present main application fields of PCM were summarized. Finally, some existing problems about PCM were discussed. This review might bring new insights to the PCM application in the BTMS.

## 2. Phase Change Material

PCM refers to a substance that could absorb or release latent heat to keep the temperature as almost constant, and what is widely used in the field of thermal management because of the special characteristics [20].

### 2.1. Classification of Phase Change Materials

**Figure 2:**
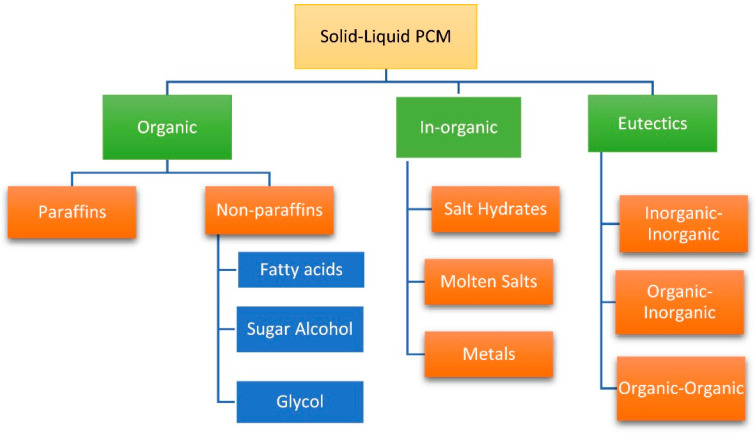
Generalized categories of phase change materials (PCMs) (Reprinted from Reference [21] with permission from Elsevier.).

There were a large variety of classification standards for PCMs. Figure 2 shows a kind of explicit classification [21]. According to the phase of substances before and after the changing process, PCM could be roughly divided into solid–solid PCMs (SSPCMs), solid–liquid PCMs (SLPCMs), solid–gas PCMs (SGPCMs) and liquid–gas PCMs (LGPCMs). Among them, SLPCMs have the characteristics of large latent heat capacity as well as the small volume changing during the phase changing process, and easy access to raw materials, which puts it within the most common application field [22,23,24]. From Figure 2, as for PCMs that undergo phase change processing between a solid and liquid status called SLPCM, they could be generalized and classified into three main categories as organic, in-organic and eutectics.

Organic PCMs mainly refer to PA and non-paraffin compounds, among which non-paraffin compounds include stearic acid, polyols, long-chain alkanes and so on. Because organic PCMs have the advantages of corrosion resistance, safety nontoxicity, good chemical durability and practically no supercooling, they have been the main raw material source of PCMs. However, they also have disadvantages such as poor thermal conductivity and easily leaking during the phase change process. Modification methods of organic PCMs are described in Section 2.3 in detail.

Inorganic PCMs generally include water, hydrated salts, melted salts, and metal [25], among which hydrated salts and metal are widely used. They have the characteristics of high latent heat capacity, non-flammability, non-leakage and relatively low cost. Due to the corrosiveness, easy phase separation and supercooling of the hydrated salts, the wide application is seriously hindered. In daily life, magnesium chloride hexahydrate (MgCl_2_·6H_2_O, MCH) is one of the most commonly used hydrated salts. It is rich in salt lakes, easy to be obtained, and contains large latent heat. It is usually used in the field of solar heat recovery [26]. In the present researches, it is mainly used in combination with EG to compensate for the disadvantages of hydrated salts and EG.

Eutectic PCMs are usually prepared by combining two or more substances with low melting temperature including organics, inorganics, or both inorganic and organic compounds, and they are crystal mixtures of several soluble components, which could be characterized by simultaneous melting and solidification, and the melting point and freezing point are usually lower than those of pure substances [27]. Because of the plenty of existing inorganic salts and organics, the eutectic types are increasing due to the combination of inorganic and organic compounds, resulting in many corresponding eutectic choices in each temperature range, which is the superior selection advantage when comparing to organic or inorganic PCMs. However, in fact, the kinds of eutectic could reach probably hundreds of thousands of, and recently the research on the heat conductivity is rather little. Due to the insufficient application, eutectic PCMs could not be described in detail in this review.

In general, under a relatively low-temperature range like from −20 to 5 °C, the commonly used PCM is organic compounds. While from 5 to 40 °C, the mainly used PCMs are organic compounds and hydrous salts. From 40 to 80 °C, the most popular PCMs are organic PCMs such as PA and aliphatic acid, which would improve the operating efficiency and service life for electrical equipment by 26% and 300%, respectively. Finally, for the relatively high-temperature range from 80 to 200 °C, organic PCM is hard to be applied, and fused salt plays a significant role.

**Figure 3:**
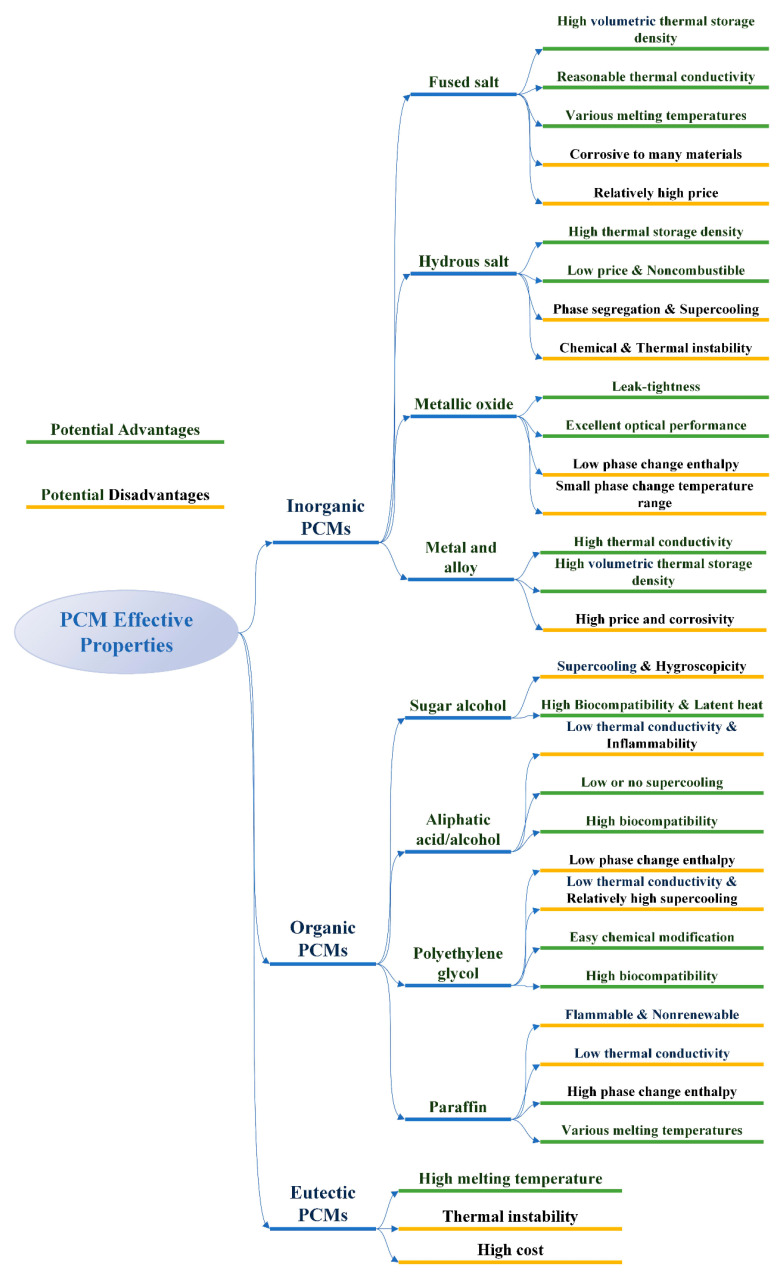
Potential advantages and disadvantages of different types of PCMs.

Figure 3 summarizes the potential advantages and disadvantages of the three kinds of PCMs.

### 2.2. Selective Conditions of Phase Change Materials

**Figure 4:**
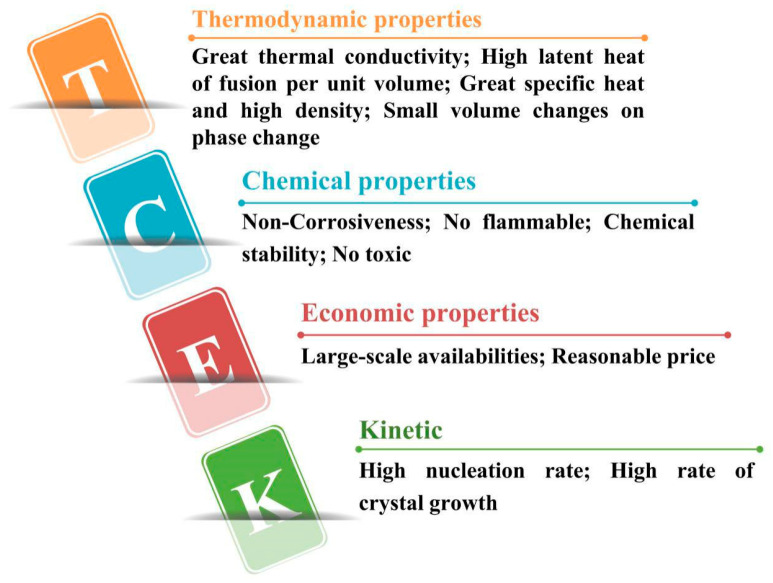
Selective criteria of PCMs (refer to Reference [28] with permission from Elsevier).

As shown in Figure 4, the main performances of PCMs in practical engineering project applications are summarized. The selection rules mainly include the following aspects:

- The phase transition temperature of PCM is within the scope of normal working temperature;
- Strong ability to absorb heat and get latent heat;
- Good thermal conductivity;
- Good chemical stability and chemical corrosion resistance;
- Low cost, easy to be obtained and not easy to leak;
- Low degree of supercooling.

### 2.3. Enhancement of Phase Change Materials with Different Improving Methods

Recently pure PCMs could not completely satisfy all the application requirements, because part of them have the disadvantages such as supercooling, low thermal conductivity and chemical instability [29,30,31]. However, except for the metal-based PCMs, other types might have the characteristic of low thermal conductivity. Among them, the thermal conductivity of organic PCMs is the lowest, but the thermal conductivity of most non-metal-based inorganic PCMs is only a bit higher than that of the organic ones [32]. Therefore, the method of improving the thermal conductivity is the main focus of researches on the performance and application of PCMs.

**Figure 5:**
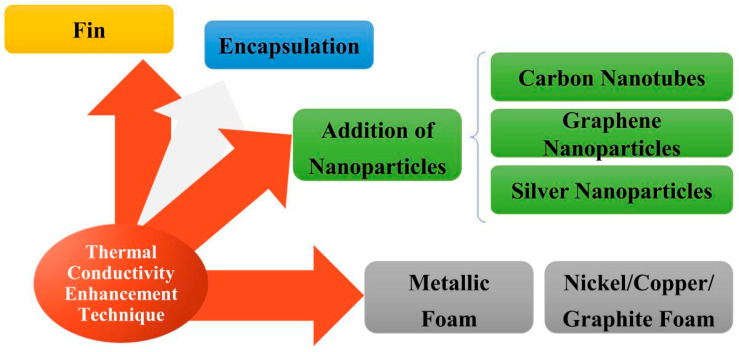
The ways about improvement for PCM thermal conductivity (refer to Reference [28] with permission from Elsevier).

With the development of PCMs, various methods have been applied to improve the thermal conductivity of PCMs, due to the increasingly strict requirements from industries [33,34,35,36,37,38,39,40,41]. From Figure 5, adding fins, packaging technology and adding high thermal conductivity fillers are the most significant methods at present. The method of adding fillers has become the main modification method due to its low cost and easy operation. In order to give readers a more systematic and comprehensive understanding, Table 2 is presented here with various methods for the improvement of thermal conductivity, illustrating the advantages and disadvantages of each way [42].

## 3. Adding High Heat-Conducting Fillers

As mentioned above, pure PCM substances would have the defect of low thermal conductivity. Researchers found that this defect could be overcome by compounding PCMs (CPCMs) with high thermal conductivity fillers, thereby improving the thermal conductivity and efficiency [43,44,45].

### 3.1. Adding Nanoparticles

#### 3.1.1. The Effect of Adding Nanoparticles on the Thermal Conductivity by Changing Structure

According to the previous researches [46,47,48], adding fillers with high thermal conductivity could change the microstructure and components of composite materials interface, thus influencing the thermal conductivity of PCMs. Colla et al. [46] added Al_2_O_3_ and carbon black (CB) to two kinds of PAs (RT20 and RT25) with different melting temperatures, and found that in the presence of 1 wt% CB nanoparticles, the thermal conductivity increased by more than 35%. In terms of the ability to increase thermal conductivity, CB showed better performance than Al_2_O_3_.

**Figure 6:**
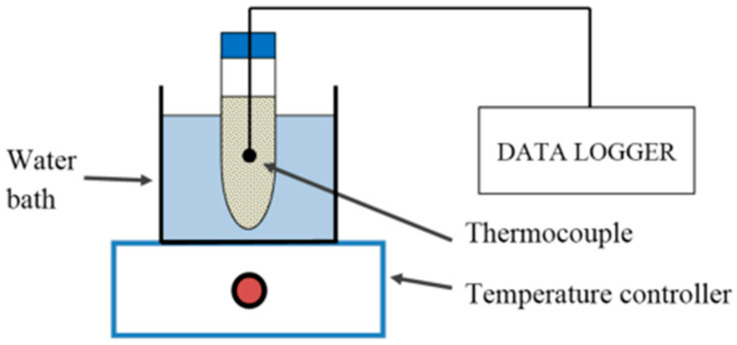
Schematic diagram of heat storage/release performance test (refer to Reference [47] with permission from Elsevier).

The low thermal conductivity of PCMs limited the efficiency with the latent heat storage. Therefore, Ramakrishnan et al. [47] studied the thermal properties of PA/expanded perlite (EP)/exfoliated graphene nano-platelets (xGnP) composite PCM by adding xGnP to PA, as presented in Figure 6. The results indicated that due to the presence of xGnP, an interconnection network was formed on the internal porous structure of EP, thereby significantly improving the thermal performance of composite PCM, and adding 1 wt % xGnP could increase the thermal conductivity by 49%. Zhang et al. [48] designed a method to prepare PCM emulsion containing n-hexadecane with the aid of an emulsifier. Compared with solid PCM, the emulsion had higher fluidity and volume change, thereby achieving better thermal conductivity. The experiments also showed that higher thermal stability and thermal conductivity could be achieved by adding SiO_2_ nanoparticles.

#### 3.1.2. The Effect of Adding Nanoparticles on Latent Heat

Besides the above properties, another important characteristic of PCM is the high latent heat performance. Although there are many concerned researchers in this field, the latent heat performance is seldom studied. Therefore, there is almost no comprehensive summary of the proposition of “what is the effect of the nanoparticles addition on the latent heat?”. In addition, there was some controversy on whether the nanoparticles addition could increase or decrease the latent heat.

For example, Colla et al. [46] used two different types of PAs (RT20 and RT25) as PCM substrates and added Al_2_O_3_ nanoparticles. The experimental results indicated that when 1 wt % of Al_2_O_3_ nanoparticles were added, the composite PCM latent heat was gained to 110% of the initial data. Elbahjaoui et al. [49] produced a type of composite PCM by adding 2–8 vol % copper nanoparticles into PCM substrate with octadecane. The experimental results showed that the latent heat of composite PCM increased due to the presence of nanoparticles. Rufuss et al. [50] improved composite materials performance by adding 3 wt % graphene oxide into PA, and found that the latent heat of the composite PCM decreased obviously. In summary, the results could be divided into two groups:

- Positive effect of nanoparticles: some studies have shown that the latent heat of composite PCM increased due to the presence of nanoparticles;
- Negative effect of nanoparticles: some studies have shown that the latent heat of composite PCM was reduced due to the presence of nanoparticles.

The reasons for the positive effect might be due to the surface charge state of the nanoparticles, the stratification of the liquid–solid phase, and the movement mechanism of phonons. The reason for the negative effect of nanoparticles might be from the arrangement of carbon–oxygen bonds in the crystal lattice, sp^2^ hybridization, dispersibility with organic solvents, volume change during the expansion of graphene oxide and so on.

### 3.2. The Effect of Adding Metal Fillers

Due to the high thermal conductivity and strong mixing ability, researchers paid great attention to materials containing metal elements. Metal materials indicate the substances with high heat conductivity and which are made with element(s) from the center and left columns of the periodic table. Commonly used fillers for PCMs include metal foams and metal particles. In addition, the presence of metal oxides could also increase the thermal conductivity of PCMs.

#### 3.2.1. Metal Foams

Metal foam is metal with a porous structure, a certain strength and rigidity, which contains a large number of pores [51]. Rangappa et al. [52] used two different methods of adding EG to PA (PCM) and immersing PCM in copper foam to explore the effects of EG and metal foam. The experimental results showed that: EG had a small mass, which was conducive to the realization of a lightweight design for thermal management, so EG was suitable to be applied in battery packs that had strict mass requirements or devices and equipment needed to lose weight; although the mass of copper foam was large, the thermal conductivity was superior, so it could be used in the thermal management system for large-scale battery packs. Zhang et al. [53] improved the thermal conductivity of PA by adding copper foam, and the results presented that the thermal conductivity of composite PCM was distinctly increased due to the excellent thermal conductivity of metal Cu foam. In addition, the irregular distribution of pores inside the copper foam restricted the liquid flow, resulting in weakening the convective heat transfer effect on the liquid phase.

Xiao et al. [54] made PA/Ni foam composite PCM and PA/Cu foam composite PCM through vacuum impregnation, respectively. From the test, by comparison with pure PA, the thermal conductivity of composite PCM was greatly improved, and that of PA/Ni foam was three times that of pure PA. In addition, due to the presence of metal foam, the phase transition temperature changed. Xiao et al. [55] studied the effect of impregnating nickel foam and copper foam with PA under vacuum on the thermal performance. Through experiments, the results showed that reducing the metal foam porosity would lead to a gain of the thermal conductivity, but the foam pore size had no remarkable effect on the thermal conductivity. Therefore, compared with pure PCM such as PA, the use of metal foam could greatly improve the thermal conductivity of composites. Thapa et al. [56] prepared a kind of composite PCM by using copper foam/eicosane wax for bench-scale heat storage. Experiment results proved that metal foam could enhance the thermal conductivity of PCM. Chen et al. [57] adopted aluminum foam to enhance the thermal conductivity of PA. Experimental data indicated that metal foam could improve the heat transfer efficiency of SLPCMs. In addition, from the experimental results, the metal foam structure had a particular obvious impact on the heat transfer process in a solid–liquid state. Wang et al. [58] studied the thermal conductivity of PA/aluminum foam composite PCM and proved that aluminum foam could improve the thermal conductivity of PA. Although the presence of aluminum foam inhibited local natural convection, it could accelerate the melting process and enhance the temperature uniformity of PCM.

**Figure 7:**
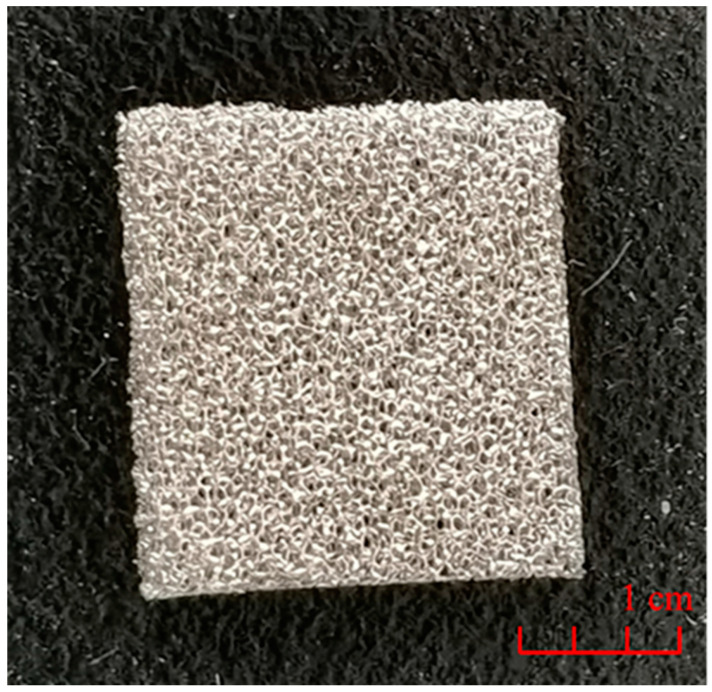
Nickel foam.

Moreover, metal foam could be used not only as a thermal conductive filler, but also as a thermal conductive substrate. Metallic nickel has good ductility, plasticity and corrosion resistance. If nickel foam is selected as a heat conductive matrix, its smooth surface could reduce the thermal contact resistance, and good plasticity could help forming enough pores, so it is very suitable to be a heat conductive matrix of PCM. The structure of nickel foam is shown in Figure 7.

**Figure 8:**
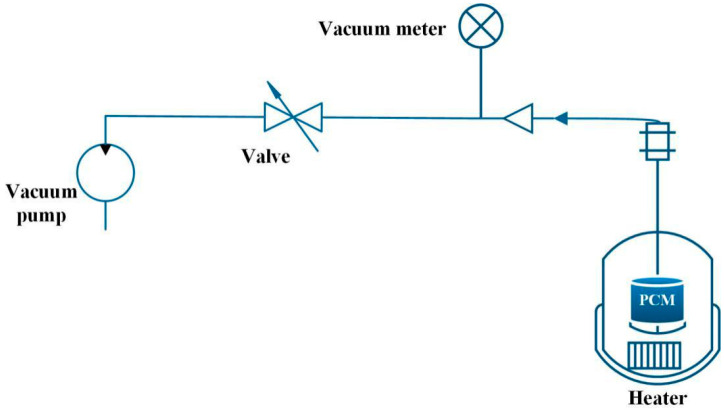
Schematic diagram of vacuum immersed PCM experiments (refer to Reference [54] with permission from Elsevier).

Xiao et al. [54] manufactured PA/Ni foam and PA/Cu foam composite PCM under vacuum and non-vacuum conditions, respectively. Figure 8 shows the vacuum impregnation process. Firstly, a net was placed for taking out the composite material at the bottom of the container. Secondly, PA and metal foam were put into the container, and the vacuum pump connected the container to the vacuum, then the vacuum in the container was carried out. PA was heated to be melted in a water bath, and the metal foam was sunk into the molten PA. The metal foam would automatically be physically immersed in the liquid PA during the sinking process. Finally, after the PA was completely solidified, the porous metal foam impregnated with PA was taken out. The experimental results showed that the thermal conductivity of PA/nickel foam was three times that of pure PA, and the thermal conductivity of PA/copper foam was fifteen times to that of pure PA. In addition, compared with pure PA material, the freezing temperature of PA/metal foam decreased, while the melting temperature increased.

Although these studies researched a relatively simple composite of PCM and metal materials with higher thermal conductivity, the thermal conductivity and structural strength could well meet the requirements of BTMS. By reasonably selecting different metal foams, significant effects could be achieved in reducing the battery packs temperature and the heat difference.

#### 3.2.2. Metal Particles

**Figure 9:**
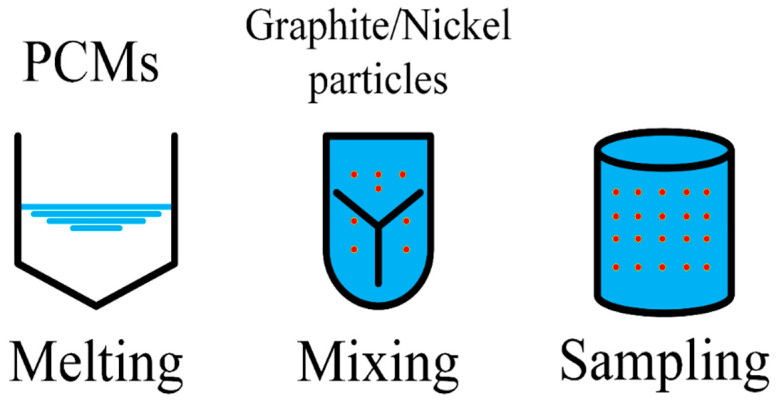
Steps to produce phase change composite (PCC) containing erythritol well mixed with highly thermal conductive graphite or nickel particles (refer to Reference [60] with permission from Elsevier).

Metal particles are widely used additives to improve the thermal conductivity of PCM. Ghossein et al. [59] used three kinds of different curing methods, i.e., ice-water bathing, room temperature and oven curing, to make nano-silver/eicosane composite PCM with different composition ratios by weight. The experimental results showed that the thermal conductivity increased with the increase of temperature, and when the external temperature approached the melting point, the thermal conductivity rose sharply. In addition, the thermal conductivity by using oven curing was the highest, followed by that from room temperature, and that by the ice-water bath was the lowest. Finally, differential scanning calorimetry (DSC) experiments were conducted. As the decreasing of the eicosane amount, the content of metal particles increased, and the latent heat and melting point of the material would decrease accordingly. Since the thermal conductivity of EG and nickel particles (NP) was two orders of magnitude greater than that of erythritol, Oya et al. [60] prepared erythritol/NP composite PCM and erythritol/EG composite PCM, respectively, as stated in the figures about the procedure for producing phase change composite (PCC) containing erythritol well mixed with highly thermal conductive nickel particles in the reference. Figure 9 illustrates the process for PCC production in a simplified sequence flow diagram. The experimental results presented that with the increase of fillers content and aspect ratio, the thermal conductivity also gradually increased. Therefore, the use of high thermal conductivity fillers could effectively enhance the thermal conductivity of the composite PCM. Cui et al. [61] discovered that nano Cu particles with high thermal conductivity could improve the supercooling and thermal conductivity of sodium acetate trihydrate through experiments. The melting and freezing experiment showed that the thermal conductivity of composite PCM was increased by nearly 20%, and the addition of 0.5% of nano-copper as the most appropriate amount reduced the supercooling degree by about 0.5 °C, which indicated that the nano-copper reducing the sodium acetate trihydrate had obvious advantages as for supercooling.

#### 3.2.3. Semimetal Materials

##### Carbon Fiber

Due to the small volume of carbon fiber (CF) [62], it could present a uniform distribution status in the PCM, and the fibrous structure could be connected to each other, so it could form a network structure with high thermal conductivity, thereby greatly improving that of the PCM. In addition, since the density of CF is quite small, it has less influence on the overall latent heat and convective heat transfer coefficients of the PCM. In addition, CF could be compounded with various PCMs.

Babapoor et al. [63] prepared PA/CF composite PCM, and studied the effect of CF length and its mass ratio on the thermal conductivity. The experiment results expressed that, due to the presence of CF, the composite thermal conductivity was significantly improved, which was affected by the length and mass fraction of the CF; in addition, the experimental results showed that 0.46 wt % and 2 mm long CF could obtain the best thermal performance; 0.46 wt % and 2 mm long CF showed the smallest temperature difference.

As shown in the figures of the operating principle of two experimental methods: the melting dispersion way and the hot-pressing way in Reference [64], Nomura et al. [64] prepared CF/erythritol composite PCM by melt dispersion and the new hot-pressing method, respectively. Due to the existence of CF, the material had a network structure with high thermal conductivity. The experiment data showed that the thermal conductivity of the material made by the new hot-pressing way was higher than that from the conventional melt dispersion means, and the preparation by the new hot-pressing required less additives. In addition, the thermal conductivity of erythritol increased from 0.73 to 30 W/(m·K) due to the presence of 25 vol % CF. Zhang et al. [65] selected two kinds of short CFs (SCFs) with different lengths mixing with erythritol to prepare composite PCM, while the composite PCM was with different mass fractions as 1%, 2%, 4%, 7% and 10% of CFs, respectively. The presence of SCFs had little effect on the melting point of the material, but the phase change enthalpy was inversely proportional to SCF content. At the same time, the thermal conductivity of the material non-linearly increased with the increase of SCF content. Tian et al. [66] selected PA as PCM substrate, CF and EG as fillers to enhance thermal conductivity, and ethylene-vinyl acetate (EVA) as supporting material to prepare composite PCM. Among them, the mutual synergy from EG and CF was helpful to enhance the thermal conductivity, and the existence of CF could reduce the leakage of PCM. The experimental results demonstrated that the thermal conductivity in the longitudinal direction were lower than that in the horizontal direction, and the thermal conductivity increased with the gain of ambient temperature.

##### Graphene Nano-Platelet

**Figure 10:**
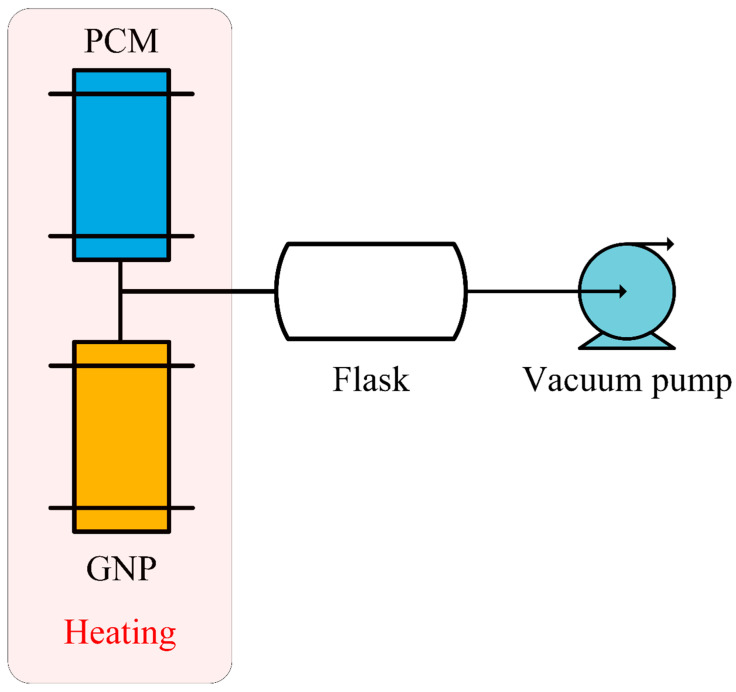
Flowchart of impregnation process (refer to Reference [69] with permission from Elsevier).

Graphene has attracted great attention from researchers due to its unique chemical and physical properties, large specific surface area, large aspect ratio and excellent thermal conductivity [67,68]. As shown in the figure about the schematic diagram of the impregnation process in Reference [69], Mehrali et al. [69] made palmitic acid/graphene nano-platelet (GNP) composite PCM with a stable shape by the impregnation method. In addition, the simplified flowchart of impregnation is as indicated in Figure 10. In the composite PCM, GNP was not only an additive to improve thermal conductivity, but also could be a supporting material to restrain palmitic acid leaking. Thermogravimetric (TG) experiments could confirm that the presence of GNP would enhance the thermal stability of palmitic acid. In addition, the thermal conductivity of composite rose by 10 times and the latent heat was improved by eight times due to the presence of GNP. In general, palmitic acid/GNP composite PCM had broad application prospects in latent heat storage fields such as power plants, solar collectors and electronic equipment. Amin et al. [70] successfully manufactured a beeswax/graphene PCM composite that could be used for building thermal energy storage (TES) by using an ultrasonic method. Through experiments, it was found that the addition of graphene led to an increase of composite PCM thermal conductivity, latent heat, heat capacity and viscosity. For example, adding 0.3 wt % graphene could increase the thermal conductivity by 11 times from the comparison to pure beeswax, increasing the latent heat by 22.5% and the heat capacity by 12%, respectively. In addition, the thermal conductivity did not always increase as the mass ratio of graphene increased. After reaching the maximum, the continued addition of fillers would no longer improve the thermal conductivity due to agglomeration phenomena. In order to enhance the thermal conductivity of PA, Liu et al. [71] prepared two composite PCMs as PA/graphene and PA/graphite flake, respectively, and the fillers content were in the range of 0–2 wt %. From experimental results, the thermal conductivity of 2 wt % PA/graphene was 58.6% higher than that of pure PA materials, of which 2 wt % PA/graphite flake was 41.4% greater, which proved that graphene was better than graphite flakes when considering the effect on improving thermal conductivity, which also demonstrated that graphene could effectively increase the thermal conductivity of PA. In addition, through experiments, it was also found that:

- Graphene and PA were tightly combined in structure without any microcracks or loose interfaces, which was also indicated by graphite flakes and PAs.
- The melting point of pure PA was higher than that of PCM composite.
- With increasing filler content as well as mass fraction, the enthalpy value of the composite PCM first increased and then decreased.

Li et al. [72] investigated the influence of sponge graphene on the latent heat and thermal conductivity of docosane/sponge graphene composite PCM. According to the experimental results, when compared with pure docosane, the latent heat and thermal conductivity of composite PCM were improved. By using scanning electron microscopy (SEM) to observe the microstructure, the existence of graphene as a nucleating agent could increase the crystallinity of composite materials, thus improving the latent heat. In addition, because of the high heat conductivity of graphene, the thermal conductivity of composite PCM has been enhanced. Therefore, optimizing the microstructure of the composite materials interface is an alternative method to increase the latent heat and thermal conductivity of PCMs. Mehrali et al. [73] studied the effect of nitrogen-doped graphene (NDG) as the filler on palmitic acid/NDG composite material. From the DSC data, when comparing to pure palmitic acid, the composite PCM had larger latent heat and better thermostability. Moreover, not only the electrical resistivity of composite PCM decreased significantly, but also the heat conductivity coefficient increased remarkably by over six times to the contrast substance. This work gives a direction for changing the latent heat and thermal conductivity of PCMs containing palmitic acid.

##### Carbon Nano-Tubes

As for the traditional organic PCMs such as PA, their poor conductivity for heat results in the slow melting and curing speed, thus their thermal properties needs to be improved during the application processing. Due to the advantages of high thermal conductivity (around 6000 W/(m·K)), low density, large specific surface area, and close density to organics, CNTs are easy to be compounded with organic matrix [74,75], thus researchers used CNTs as fillers in PCMs to enhance the thermal conductivity. The presence of CNTs could increase the thermal conductivity of composite PCM by nearly one order of magnitude. At present, there are two main types of CNTs: single-walled carbon nanotubes (SWCNTs) and multi-walled carbon nanotubes (MWCNTs), of which MWCNTs are more widely used [76,77], and scanning electron microscopy (SEM) results showed that MWCNTs could be more uniformly dispersed in PCMs.

Ye et al. [78] prepared a composite PCM of Na_2_CO_3_/MgO/MWCNT, in which Na_2_CO_3_ was used as the substrate, and MgO was used as the support material, while MWCNT was applied as the filler to improve the thermal conductivity of composite PCM. The SEM results showed that the MWCNT was uniformly dispersed on the Na_2_CO_3_/MgO composite PCM. In addition, with the increased content as mass fraction of MWCNT, the thermal conductivity of the material increased correspondingly at a temperature of 120 °C, and the addition of 0.5 wt % MWCNT led to a rise in thermal conductivity of 69%, while temperature also had the similar effect. Xu et al. [79] prepared PA/diatomite /MWCNT composite PCM, in which diatomite was used as the support material, while PA and MWCNT were used as PCM and filler, respectively. Through experimental analysis, diatomite calcined at 600 °C for 2 h had the best performance, while the heat treatment would not change the absorption capacity of diatomite earth for PA, so this kind of diatomite was selected as a filler for the composite PCM. In addition, the material showed good chemical compatibility, thermal conductivity and thermal stability, and the heat storage/dissipation rate was enhanced due to the presence of fillers.

**Figure 11:**
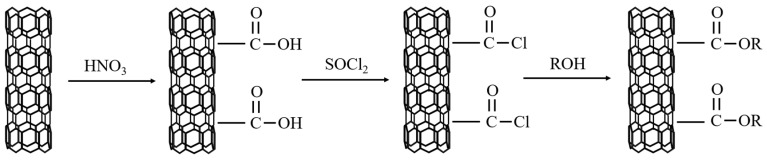
The flow diagram with the formation reaction steps of grafted CNTs.3.2.4 Metal oxide.

As shown in the figure about the sketch map of the formation reaction steps of grafted CNTs in Reference [80], Li et al. [80] grafted CNTs with polyols such as octyl alcohol, tetradecyl alcohol, and stearyl alcohol under acidic conditions. Figure 11 illustrates the reaction steps for grafted CNTs production. The grafted CNTs were used as fillers and PA was used as PCM substrate to prepare composite. According to the analysis of experimental data, the CNTs grafted with polyols had greater dispersibility than ordinary CNTs, which were shortened and reduced aggregation, so the ability to enhance thermal conductivity was significantly improved. At the same time, the grafting reaction was carried out among the three kinds of polyols, and the performance of grafting with stearic acid was the best, since the grafting rates of octanol, tetradecyl alcohol, and stearyl alcohol were 11%, 32%, and 38%, respectively. Li et al. [81] used stearyl alcohol to graft CNTs and prepared grafted CNTs with stearyl alcohol to acquire (CNT-SA)/PA composite PCM. After 100 high temperature/cooling cycle experiments, the material still had good durability and thermal stability. In addition, the milled CNTs had good dispersibility and thermal conductivity. Only 4 wt % CNT could increase the thermal conductivity of PCM by 79.2%.

**Figure 12:**
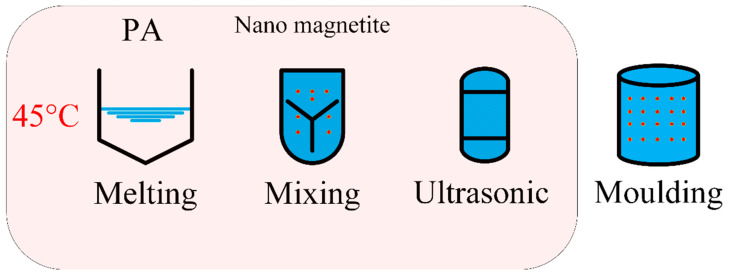
Processing steps of dispersion technique (refer to Reference [82] with permission from Elsevier).

Although the thermal conductivity of metal oxide materials is significantly poorer than that of metals, its thermal conductivity is much higher than that of most PCMs. Therefore, researchers selected some metal oxides as additives to enhance the thermal conductivity of PCM. Sahan et al. [82] prepared PA/nano-Fe_3_O_4_ composite PCM by mixing nano-Fe_3_O_4_ by adding 10 wt % or 20 wt % sol-gel method into PA, respectively. The processing steps of the dispersion technique are shown in Figure 12. DSC results indicated that the latent heat of composite PCM was 8% higher than that of pure PA. In addition, due to the presence of 10 wt % Fe_3_O_4_ or 20% Fe_3_O_4_, the thermal conductivity increased by 48% and 60%, respectively. Babapoor et al. [83] prepared composite PCM by adding SiO_2_, Al_2_O_3_, Fe_2_O_3_, ZnO and the mixtures into PA and mixing them. Due to the presence of metal oxide particles, the thermal conductivity was significantly improved.

### 3.3. Adding Non-Metallic Fillers

Besides metal materials, some researchers also considered whether inorganic carbon materials could be used as additives to enhance thermal conductivity. Currently, substances such as EG have been investigated that could be used as additives through experiments [84].

#### Expanded Graphite

Ling et al. [85] studied the factors such as mass fraction of EG, density and temperature of composite PCM that influencing the thermal conductivity of RT44HC/EG. According to the figures of experimental data diagrams with heat capacity curves and thermal conductivity curves in Reference [85], the experimental results showed that:

- As the mass fraction and bulk density of EG increasing, the thermal conductivity of the composite PCM also increased, and the maximum value could be increased to 60 times higher;
- The thermal conductivity of the composite PCM was only related to the phase transition temperature with a range from 40 °C to 45 °C, and almost doubled after the modification, but there was nearly no change for thermal conductivity under temperatures beyond the range;
- With the increasing EG mass fraction, the specific heat capacity and specific latent heat of composite PCM decreased.

## 4. Fins

Inserting metal fins into PCM is also an effective way to enhance heat transfer performance. Fins are widely used in cooling technology of electronic equipment [86,87,88] and TES [89,90,91] due to the advantages such as simple structure, easy manufacturing and significant improvement of heat transfer performance.

**Figure 13:**
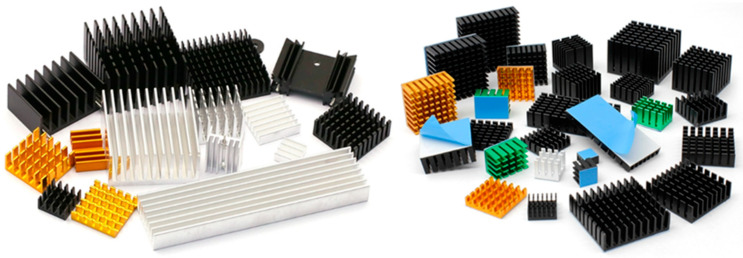
Commonly used fins in BTMS.

In order to increase the contacting area, there are two main ways to install the fins: one is to insert the fins into the PCM; the other one is to put the fins on the surface of the PCM, then the contact area between the air and the fins could be added. The essence of both is to transfer the heat absorbed by the PCM to the fins, thereby transferring the heat of the fins to the atmosphere environment to achieve the purpose of cooling. According to previous researches, the influence of fins on the heat dissipation performance of PCM-based BTMS was mainly determined by the material [92], number [93,94,95,96], length, structure [89,97,98] and other parameters. The structures of commonly used fins are shown in Figure 13.

Weng et al. [99,100] studied the influence of fins structure on the heat dissipation performance of BTMS. Through experiments, it was found that the longitudinal structure of the fin was beneficial to dissipate the heat accumulated at the bottom of the battery pack, while the circular structure of the fin could be better contacted and dissipate heat due to the larger surface area, and had good heat dissipation performance when only considering structural factors.

**Figure 14:**
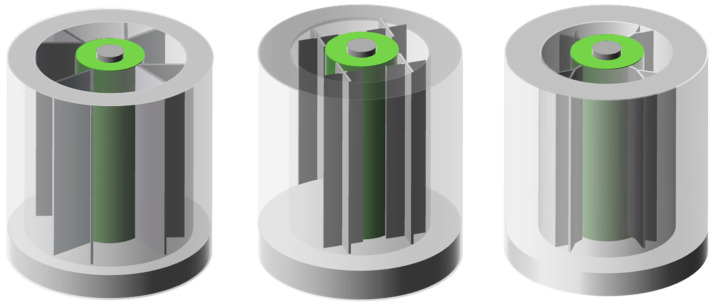
Improved modules with PCM and fins in different shapes (refer to Reference [100] with permission from Elsevier).

Based on the improved new module (Figure 14), from experimental results, the heat dissipation performance of these new structured fins was generally higher than that of rectangular fins, which enhanced the temperature uniformity in BTMS. In addition, the results also showed that: (a) The battery pack generated more heat in a high-temperature environment than in a room-temperature environment; (b) Under high-temperature environment, the capacity of the battery decreased obviously and the aging phenomenon was obvious; (c) As for the heat flow path, more fins did not show the better efficiency. While the cost, heat dissipation efficiency and other factors should also be considered.

Zhao et al. [101] investigated the PCM melting properties in an annular space with constant inner and outer tube wall temperature by using fins and metal foams such as nickel, aluminum and copper. The results showed that the melting time first decreased and then increased with the increase of the fins quantities when the fin thickness and volume were fixed. By comparison to pure PCM, the melting time would be further decreased by 8% by appropriately increasing the fins length at the bottom. In addition, by comparing the results of using different metal foams, the fins and the metal foams had the same effect.

**Figure 15:**
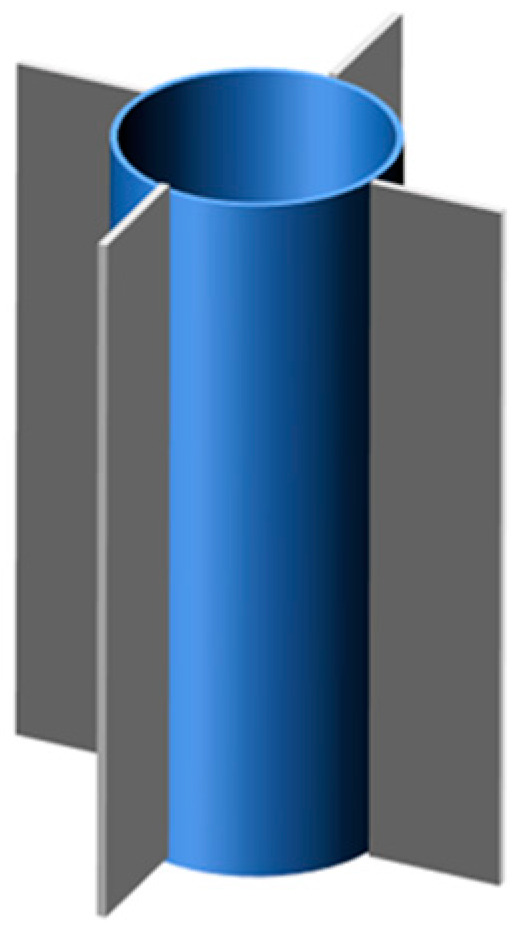
Fins with ring (refer to Reference [102] with permission from Elsevier).

Based on the defect of low thermal conductivity of PCMs, Sun et al. [102] proposed a new structure consisting of a longitudinal heat sink and ring. The sketch map of fins with a ring is as shown in Figure 15. Through experimental results, due to the presence of fins, there was a heat conduction network inside the battery, thus increasing the heat transfer area, which was conducive to the heat dissipation of BTMS. In addition, the normal working time of the battery increased with the increase of the number of longitudinal radiators, and the maximum fins qualities were eight pieces.

**Figure 16:**
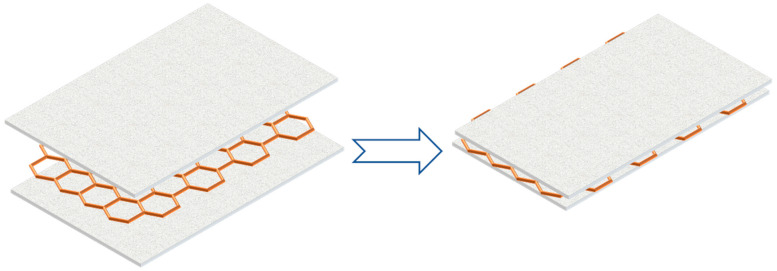
Using copper mesh to improve PCM thermal conductivity (refer to Reference [14] with permission from Elsevier).

Besides adding fins, metal mesh could also be applied to PCMs since metal is a good conductor of heat. Wu et al. [14] developed a method to improve the thermal conductivity for PA/EG composite by using copper mesh, as Figure 16 indicated. In the experiments, the copper mesh was embedded in the PCM to achieve the purpose of rapid heat transfer. In addition, the existence of copper mesh could also improve the strength of the system. Through experimental analysis, at a discharge rate of 5C, the maximum temperatures of the copper mesh structure and the copper-free mesh structure were 61.6 °C and 65.5 °C, respectively, which meant that the copper mesh structure had better heat dissipation performance than the copper-free mesh structure.

## 5. Packaging

At present, the commonly used PCM packaging technologies mainly include dispersed/decentralized packaging and microcapsule packaging.

### 5.1. Dispersed/Decentralized Packaging

**Figure 17:**
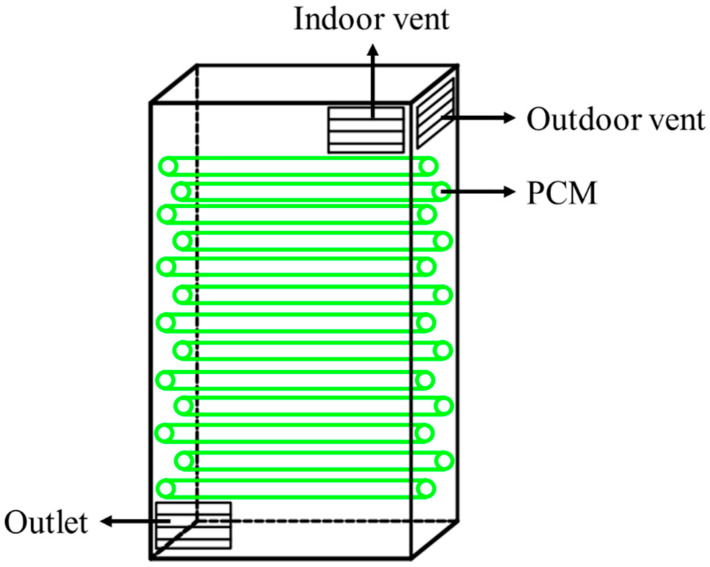
Dispersed/decentralized packaging.

Nowadays, the most widely used packaging technology in industries is the dispersed/decentralized packaging technology, which is mainly prepared by adding a certain amount of PCM to a container that would not react with PCM, and air is usually used as the heat transfer medium. In residential buildings, PCM in the form of dispersed/decentralized packaging with smaller sizes are usually mixed to meet the energy storage requirements. In addition, as shown in Figure 17, dispersed/decentralized packaging is often applied to energy storage water heaters.

### 5.2. Microcapsule Packaging

**Figure 18:**
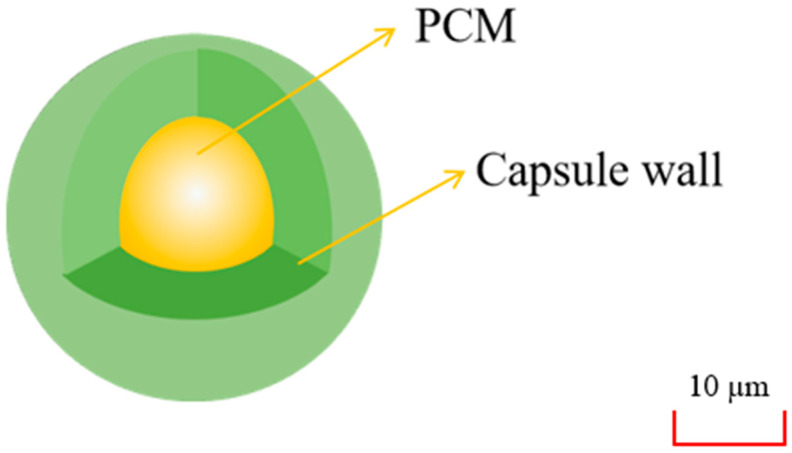
Microcapsule packaging.

As shown in Figure 18, the microcapsulation refers to a technology that with a stable polymer film coated on the surface of SLPCM particles to form a kind of PCM with a core-shell structure. The average particle size of a single microcapsulation is 1–100 µm. Because of the deficient thermal conductivity of pure PCM, the absorbed latent heat could not be released in time, so the researchers conducted related experiments on the thermal performance of microencapsulated PCM (MPCM). From previous studies, although the sizes of the material particles were small enough, the overall heat dissipation area was increased, which solved the problem of condensation on the container wall or the edge of the capsule wall when the PCM underwent a liquid-solid phase transition. At present, organic PCM such as PA and fatty acid are the main targets for microencapsulation.

MPCM could be prepared by polymerization methods through emulsion, interfacial way or other means. The material usually consists of two parts: PCM as the core and organic polymers or inorganic compounds as the shell, among which inorganic materials are often used as the outer shell of MPCM because of their high thermal conductivity. Compared with ordinary PCM, MPCM has higher thermal conductivity, so microencapsulated technology is able to significantly enhance the thermal conductivity [103,104,105]. Some enhancements for increasing the MPCM thermal conductivity have been collected, as listed in Table 3.

Yu et al. [106] used a self-assembly way to prepare an MPCM with n-octadecane as the core and CaCO_3_ as the shell. From SEM, the product of MPCM had an obvious core-shell structure. At the same time, TG experimental analysis showed that due to the existence of a CaCO_3_ shell with high thermal conductivity, that of MPCM had been significantly improved. With the protection of a CaCO_3_ shell, the resistance to penetration and service life had also been improved. In addition, MPCM also showed good thermal storage capacity. Due to the low cost and easy availability of CaCO_3_, the packaging technology had a high degree of feasibility and good application prospect.

Zhang [111] et al. designed an MPCM with n-octadecane as the core and SiO_2_ as the shell. The SiO_2_ shell was prepared by the sol-gel method using TEOS, and TEOS was the precursor of SiO_2_. SEM images proved that MPCM presented a distinct spherical shape with a clear core-shell structure. In addition, from experiment results, under acidic conditions with the pH of 2.45, the synthesized MPCM had a smooth and dense surface. TG experimental results showed that the MPCM was degraded in two steps and had good thermal stability. In addition, due to the presence of a SiO_2_ shell with high thermal conductivity, MPCM was also significantly improved.

Zhang et al. [109] designed an MPCM with n-dodecane as the core and rare-earth-doped zirconia as the shell. The shell had enhanced photoluminescence and heat storage properties. From SEM and transmission electron microscope (TEM) results, the developed MPCM exhibited a smooth surface, a regular sphere structure with uniform size distribution, and an obvious core-shell structure, respectively. Due to the existence of the zirconium shell, the thermal conductivity was increased to 0.906 comparing to 0.152 of n-dodecane, which was nearly six times higher. In addition, MPCM also exhibited good thermal regulation capability and energy storage efficiency. On the other hand, as for the photoluminescence characteristics, due to the presence of the rare earth-doped shell, the emission intensity of the blue up-conversion fluorescence and the purple down-conversion fluorescence were significantly enhanced under the excitation radiation with wavelengths of 850 and 280 nm. Peng et al. [110] prepared MPCM with the core of stearic acid and the shell of montmorillonite/smectite. The researchers measured the thermal conductivity, and the experimental results proved that the montmorillonite/smectite shell had the ability to improve it.

Many researchers chose an inorganic material shell to optimize the MPCM in order to further enhance the thermal conductivity. Wang et al. [111] prepared MPCM by using PA (RT42) as the core and calcium carbonate as the shell by adding EG and mixing them under a pressure of 10 kN. However, there was surface tension and pressure changing between MPCM and EG, but without chemical reactions. Experimental results showed that after adding 24 wt % EG, MPCM had a carbon network structure, then the thermal conductivity of it was 24 times that of RT42.

Besides inorganic materials, organic polymers have been also commonly used as MPCM shells. Whereas polymers generally have poor thermal conductivity, which have been usually modified to enhance the thermal conductivity before use. Al-Shannaq et al. [112] prepared MPCM with PA (RT21) core and PMMA shell, using polydopamine (PDA) for surface activation process and electroless silver plating to improve the thermal conductivity. The experiment focused on the effects of silver-plated coatings with different sizes and coverage on MPCM thermal conductivity. The experimental results were as follows:

- When the size of MPCM without silver plating decreased, the thermal conductivity also decreased;
- The thermal conductivity of silver-plated MPCM increased with the increase of size;
- The thermal conductivity of MPCM increased with the gain of the silver-plated coating coverage.

Jiang et al. [113] prepared MPCM by emulsion polymerization with PA core and PMMA shell. Nano-Al_2_O_3_ with high thermal conductivity was embedded in the shell to modify the materials and improve the thermal conductivity. TG analysis showed that compared with unmodified MPCM, the presence of Al_2_O_3_ made it have good thermal stability, and the thermal conductivity of modified MPCM was positively correlated with the content of nano-Al_2_O_3_, but there was a threshold for the nano-Al_2_O_3_ content, since exceeding the threshold value would reduce the phase transition enthalpy. Yang et al. [114] prepared a kind of MPCM with n-octadecane core and PMMA shell. By adding silicon nitride powder to achieve the modification purpose, a new type of MPCM was prepared. SEM images showed that the MPCM had a clear core-shell structure and a regular spherical shape. Through experimental results, the modified MPCM had high heat storage capacity, thermal conductivity increasing by 56.8% and thermal stability.

## 6. Phase Change Materials Application in Battery Thermal Management System

In recent years, based on the general trend of environmental protection and new energy development, PCM has been developed rapidly. With its remarkable feature of keeping the temperature as constant during the phase changing process, PCM is commonly applied for solar energy storage, building energy storage, electronics, thermal equipment management [115,116,117,118,119] and other related fields.

### 6.1. The Necessity of Phase Change Materials Application in Battery Thermal Management System

Due to its excellent performance, LIBs are currently one of the main power sources for HEVs and EVs [120]. However, a large amount of heat would be generated when the battery pack is discharged in normal operation. If there is no good thermal management system to facilitating the heat release in time, a major safety accident would possibly occur [121]. Therefore, it is necessary to design a corresponding thermal management system for the battery pack.

The passive thermal management system could control the battery temperature in an appropriate range, reduce the temperature difference between the batteries in the pack, thereby improving the cycle life of the battery. In addition, extra equipment such as fans or pumps are not necessary to be added into the passive thermal management system, which greatly simplifies the thermal management system and reduces the cost of the system. More importantly, in extreme working environments such as high discharge current and ambient temperature, compared with air cooling, passive BTMS could make the battery temperature lower and the temperature difference smaller [122]. Therefore, the study of passive BTMS is of great significance to promote the development of efficient and clean new energy vehicles.

In addition, the service temperature of the battery directly affects its safety performance and cycling life, while the cycling life of the battery is related to its economic performance. Previous studies have shown [123,124] that high-temperature environments could easily lead to the formation and growth of the solid electrolyte interface (SEI) inside the battery, the increase of anode resistance and the attenuation of active materials, which led to an increase in battery internal resistance and a decrease in capacity and electric power. On the other hand, low-temperature conditions might lead to the formation of a lithium metal coating inside the battery and react with the electrolyte [125], resulting in uneven current and voltage distribution in the battery. Therefore, BTMS was indispensable for improving the safety and cycle life for batteries. Table 4 summarizes some research literature on the relationship between the capacity degradation mechanism and the cycle temperature of LIBs, which also shows whether a relatively high or low temperature would accelerate the aging process of the battery.

### 6.2. Traditional Battery Thermal Management System without Phase Change Materials

BTMS usually consists of two parts: heating system and cooling system, and the current research mainly focused on the battery cooling system. There were mainly three types of traditional BTMS: air cooling, liquid cooling and heat pipe cooling.

**Figure 19:**
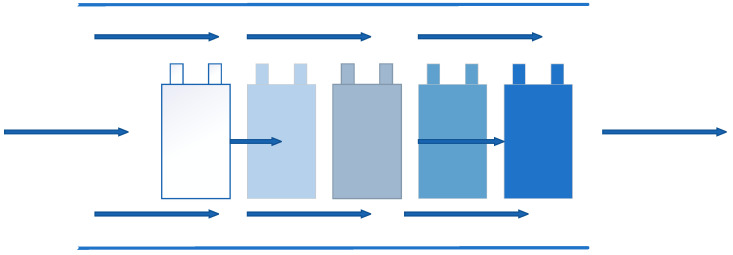
Schematic diagram of serial ventilation.

**Figure 20:**
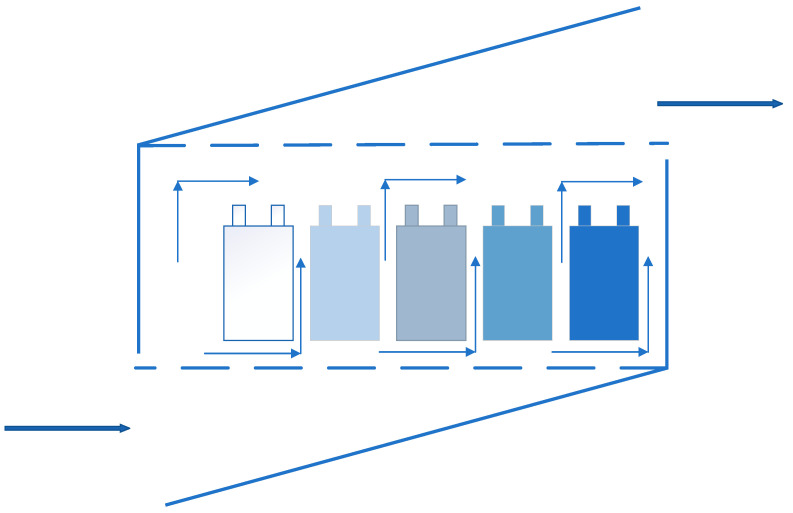
Schematic diagram of parallel ventilation.

Air cooling is the most common used cooling way, which means applying the cold air flow to take away the heat in the system, and it was the first applied method in BTMS. The National Renewable Energy Laboratory (NREL) [131,132] in the United States had evaluated the heat dissipation performance of the power battery packs inside the Insight and Prius EVs, respectively. From the tests, although the power consumption of Prius battery packs was three times that of Insight battery packs, the temperature increase of the two samples after the test was similar, which was from the result of using different ventilation structures. Compared with the serial ventilation structure, the parallel ventilation structure could keep the pressure difference of the air flowing through the gaps between the battery modules uniform, and evenly distributed the air flow through the batteries. So that the heat generated by each battery module could be taken away in time and the temperature difference was reduced. Figure 19 and Figure 20 are the schematic diagrams of serial ventilation and parallel ventilation structures, respectively.

Compared with the air cooling method, liquid cooling has a higher convection heat transfer coefficient. Therefore, the application of liquid cooling to the thermal management of batteries could better meet the requirements of high thermal load. Liquid cooling was applied in the “Ovonic” nickel metal hydride battery module produced by General Motors.

**Figure 21:**
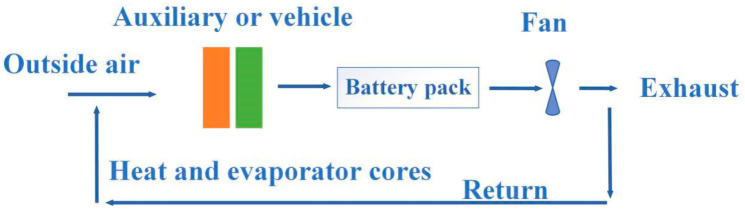
Schematic diagram of air cooling system.

**Figure 22:**
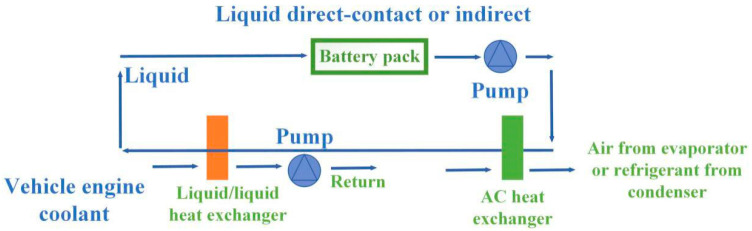
Schematic diagram of liquid cooling system.

Although air cooling and liquid cooling have proven to be effective heat management methods, both of them have great disadvantages. Figure 21 and Figure 22 show the schematic diagrams of air cooling systems and liquid cooling systems, respectively [133]. From the figures, pumps, fluid circuits, control circuits and other devices as well as additional power input were necessary in both types of cooling ways, which greatly increased the complexity and manufacturing cost of the system. Compared with the PCM-based cooling method, air cooling and liquid cooling had no obvious advantages in practical applications.

The working principle of heat pipe cooling has been explained in detail in the article by Yang et al. [134]. Although the heat pipe was known as one of the super thermal conductors, because the shape of the heat pipe could not be well matched with the battery shape, not all battery packs could be cooled by the heat pipe.

### 6.3. Phase Change Material-Based Battery Thermal Management System

Compared with the previous three kinds of traditional cooling ways, the PCM-based cooling method has gradually been the primary choice for BTMS due to the characteristics of no additional equipment, simple operation, and low cost. The large phase change latent heat enables PCM to absorb and dissipate heat to make the group stay within a safe working temperature range for a long time.

In order to verify the advantages of BTMS with PCM, Sabbah et al. [135] compared the effect of passive cooling based on PCM with forced air cooling. Passive cooling based on PCM used graphite composite PCM to cool and dissipate heat around the battery pack, while forced air cooling used fans for cooling and heat dissipation. The experimental results showed that the BTMS using PCM exhibited better cooling and heat dissipation effects under conditions such as high discharge rates and high temperature environments. On the other hand, from the forced air cooling experiments, air cooling was not a suitable BTMS, which could not consume a large amount of fans, so it was hard to keep the battery temperature at a normal working level. At the same time, the use of PCM could reduce the temperature difference between individual batteries more effectively.

Based on the rapid development of PCM over the years, various excellent composite PCMs were gradually used in BTMS for power battery packs by researchers. For example, Kizilel et al. [136] studied the performance of BTMS based on PA/graphite composite PCM in the lithium battery module, and the experimental results showed that it could effectively provide a solution to the battery overheating problem, and could control the temperature within 45℃ when the battery was discharged. In Reference [136], there was a figure showing the schematic of the experimental setup for measuring the battery heat generation rates. In addition, at the temperature of 45 °C and the discharge rate of 2.08 C, it could safely discharge at high current, and the fading rate of the battery capacity was reduced by half. Moreover, the compactness of packaging not only reduced the volume occupied by packaging and the related complex cooling system, but also decreased the total weight of high-power applications. Compared with the complex traditional cooling system, PCM components provided BTMS with superior compactness and reduced the weight and manufacturing cost of BTMS. If necessary, PCM-based passive cooling could complement or assist in performance with active cooling, so the design cost would be greatly reduced and more economical.

With the rapid development of PCM in recent years, researchers have also investigated its effect on practical application. Duan et al. [137] studied two different kinds of PCMs designed at different ambient temperatures: one was to install a PCM cylinder around the heater, and the other was to use the performance of a PCM sheath to wrap the heater. The experimental results showed that both of the two types PCMs had a positive effect of keeping BTMS within normal temperature range. In addition, the temperature of the battery could be effectively controlled under both constant and non-constant heat release rates (HRRs), and the cooling effect was better than the natural cooling method.

Although PCM has excellent performance of providing rapid temperature response and effective temperature control, there are still some shortcomings that could not be ignored, such as poor thermal conductivity, easily leaking, and low strength. Researchers usually used methods such as adding the high thermal conductivity of fillers into the PCM to remedy the disadvantages, while the specific methods have been described in detail in the above sections.

Before designing the BTMS, it is necessary to estimate the heat release of the battery pack to obtain the corresponding PCM consumption. There are three ways for the generation of heat in the battery [138]: heat released from the chemical reaction inside the battery, heat from the internal resistance, and heat from the polarization. Mills et al. [139] experimentally found that by selecting a PCM matrix impregnated with EG, the problem of low thermal conductivity could be significantly improved. The simulation results showed that although the total heat produced by the battery was accounting for only a small portion of the electric energy, the heat released would cause the temperature to rise rapidly to a level that was unfavorable to the battery and became a safety issue. In addition, even at high discharging rates, the volume of the battery pack and the number of PCM could be increased to make the temperature meet the standard requirement of below 55 °C.

According to the heating power of the battery and the required temperature control time, the total heating value of the battery could be obtained by multiplying the power and the required time. According to the calorific value of the battery, the mass of PCM required by BTMS could be calculated as follows:

Heat production of the battery is equal to the sensible heat of the PCM plus latent heat of the PCM and convection heat dissipation [140,141,142].

The convective heat dissipation is relatively small, so it could be omitted. The above definition could be expressed by mathematical expression as follows:

*Q* = *m c_p_* ∆*T***+***m H* (1)

**Figure 23:**
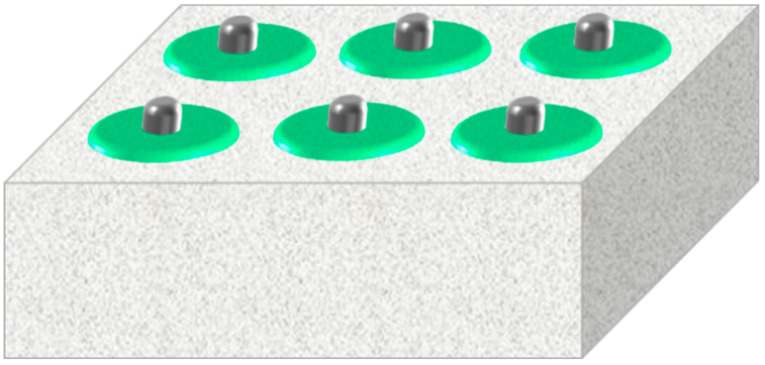
Schematic diagram of battery pack cooling module.

**Figure 24:**
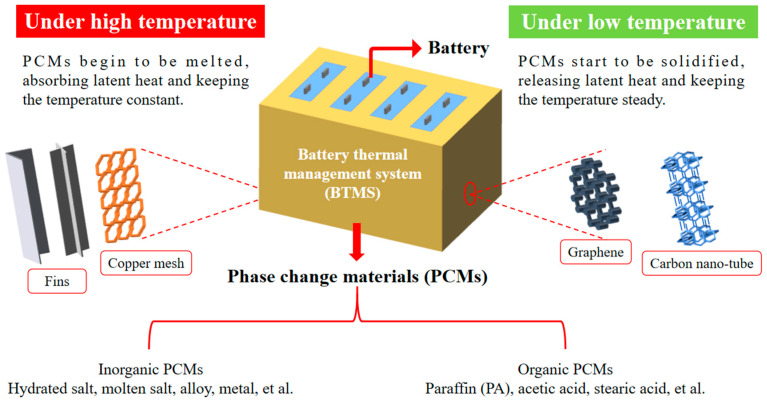
Operating principle of PCMs with thermal conductivity improvement methods such as adding fins and fillers application in BTMS under various temperatures.

In the formula, *Q* is the heat produced by the battery, *m* is the mass of the required PCM, *c_p_* is the specific heat capacity of the PCM, ∆*T* is the temperature difference in the PCM, and *H* is the phase change enthalpy of the PCM. Khateeb et al. [143] calculated that each 18,650 LIB with a capacity of 2 Ah needed 12 g of PA to absorb heat by using Formula (1). Since the volume of PCM would increase correspondingly after melting, it was necessary to reserve 10% of the volume in the system. Therefore, the required mass of PCM could be calculated, and then the geometric size of the PCM matrix could be determined according to the shape of the battery. Finally, holes of the same size as the battery were uniformly dug out in the PCM matrix. The number of holes was determined by the number of batteries in the battery module. The final battery module is as shown in Figure 23. In this review, the working principle of PCM with thermal conductivity improvement methods such as adding fins or fillers applied in BTMS under high or low temperatures is summarized and illustrated in Figure 24.

## 7. Conclusions

This review mainly summarizes and evaluates the current application status of PCM and the significant development direction. The conclusions are as follows:

- In terms of enhancing the PCM thermal conductivity, although the heat dissipation capacity of the system could be greatly improved by using metal fins, it would increase the system mass and the manufacturing cost to a certain extent. However, the method of adding fillers could only increase the thermal conductivity of PCM within a limited range, and it is necessary to collect relevant knowledge and comprehensively understand the mechanisms in the preparation stage. Thus, the threshold is high and it is necessary to choose the appropriate method reasonably in practical application.
- Passive thermal management system with PCM has the advantages of simple structure and low manufacturing cost. However, it is difficult to meet the needs when charging and discharging large battery packs, so it is mostly used for a small battery/cell pack. An active thermal management system based on PCM, which has complex structure and high manufacturing cost, is obviously more superior than the passive one in heat dissipation capacity, and the active one is very suitable for large-capacity battery packs. In practical application, a reasonable choice is needed to be considered.
- The structure of the BTMS with PCM needs to be further optimized to ensure the safety of the battery pack. In the meantime, the production cost and actual volume should be reduced, so that it could improve the safety of the battery efficiently.
- Making full use of the PCM characteristics could realize the design of a thermal management system that effectively recycles battery heat under supercooling conditions.

In this review, methods about improving thermal conductivity for PCMs were summarized in detail, while there are still some technology problems and challenges to be faced in the future, such as supercooling, which is a main problem that could influence the thermal performance and stability of PCMs, and it is necessary to be investigated and improved further. In addition, hydrous salts are strongly caustic and could probably destroy the structure of other materials, which would affect the service life of the battery pack and buildings. In addition, due to the unbalanced relationship between the demand and supply, the average price of PCMs is relatively high. However, PCMs have important market potential in the fields of buildings and energy management, and the application would be more widespread in the future. Therefore, how to prepare PCMs with lower cast is the main problem that needs to be solved.

In summary, this review mainly introduces the methods and applications for improving the thermal conductivity of PCM, and then puts forward opinions on the development of PCM. In addition, researchers also need to develop and study new methods or new PCMs to meet the requirements of BTMS.

## Figures and Tables

**Table 1:** The list of the accidents caused by thermal runaway (TR) of LIBs in recent years.

Time	Location/Fire Source	Cause
26 April 2015	A power station in Shenzhen City	Overcharging of batteries caused electrolyte leakage in several storage boxes, resulting in the short circuit of batteries and a fire.
14 May 2016	A bus station in Zhuhai City	A large-scale battery short-circuit caused the spontaneous combustion.
24 August 2016	Samsung Galaxy Note 7	Due to the design defect of the battery in the phone, a short circuit occurred, causing spontaneous combustion.
27 September 2017	Newman Company, Shenzhen City	Due to the short circuit, batteries stored in a warehouse caused spontaneous combustion.
30 November 2017	A company in Dongxihu District, Wuhan City	Rainwater penetrated into the batteries and caused chemical reactions, resulting in the spontaneous combustion.
31 May 2018	A rental in Chancheng District, Foshan City	Batteries in an electromobile were short-circuited because of charging for a long time, causing a fire.
25 February 2018	Flight CZ3539 from Guangzhou Baiyun Airport to Shanghai Hongqiao Airport.	Spontaneous combustion caused by a power bank.
11 June 2019	A travel agency in Dali City	LIBs caught fire during the charging process, igniting the surrounding combustible materials, expanding to a major fire accident.
8 May 2020	A car in Tangxia Town, Dongguan City	Spontaneous combustion of LIBs caused a fire.
16 August 2020	An electric vehicle (EV) in Taiyuan City	Spontaneous combustion of LIBs inside the electric car during charging.

**Table 2:** Summary and comparison of the advantages and disadvantages of three ways for improvement of PCM thermal conductivity.

Ways	Advantages	Disadvantages
Adding fins	Increasing high efficiency of heat dissipation; simplifying the operation process; obtaining available materials easily.	Being with poor refill ability; having large contact thermal resistance; being with high cost; having large volume.
Adding fillers	Being with low cost; improving the latent heat; obtaining available materials easily.	Being easy to be aggregated and precipitated; having insufficient thermal uniformity in BTMS.
Encapsulation	Being with corrosion resistance; having good strength and great flexibility; having superior sealing performance; having high safety.	Being with high technological demand; being with high requirements for packaging materials.

**Table 3:** List of different packaged PCMs effect on thermal conductivity.

PCM Core	PCM Thermal Conductivity k_p_ (W/m·K)	Shell	Shell Thermal Conductivity k_a_ (W/m·K)	Encapsulation Efficiency (%)	Encapsulated PCM Thermal Conductivity k_c_ (W/m·K)	Magnification (Times)
n-octadecane [106]	0.15 (solid phase)	CaCO_3_	2.47	40.04	1.26	8.26
n-octadecane [105]	0.15	SiO_2_	1.30	57.70	0.62	4.13
n-octadecane [107]	0.15	ZrO_2_	2.56	64.52	0.91	5.96
PA (RT42) [108]	0.37	CaCO_3_	-	-	0.81 8.86 (with 24 wt % EG)	2.21 24.00
PA [109]	About 0.26	SiO_2_	-	50.80 49.60	1.03 1.16 (graft with graphene oxide)	3.89 4.38
PA (RT21) [110]	0.15	Polymethyl methacrylate (PMMA)	0.19	-	0.19 2.41 (coated with silver)	1.26 16.00

**Table 4:** The relationship between the capacity fading/attenuation of LIBs and operating temperature.

Authors	Materials	Discharge Interval	Cycling Rate	Cycles	Cycling Temperature/°C	Capacity Fading/Attenuation
Zhang et al. [126]	C/LiFePO_4_	3.6~2.0 V	3CC/1	600	45 25 0 −10	25.6% 14.3% 15.5% 20.3%
Liu et al. [127]	C/LiFePO_4_	90.0% DOD	C/2	757 2628	60 15	20.1% 7.5%
Amine et al. [128]	Meso carbon microbcads (MCMB)/LiFePO_4_	3.8~2.7 V	C/3	100	55 37 25	70.0% 40.0% small
Shim et al. [129]	C/Li[Ni_0.8_Co_0.15_Al_0.05_]O_2_	100.0% DOD	C/2	140	60 25	65.0% 4.0%
Ramadass et al. [130]	C/LiCoO_2_	4.2~2.0 V	C/9~C/1	300	55 25	26.7% 10.1%

## Abbreviations

BTMS	battery thermal management system
CB	carbon black
CF	carbon fiber
CM	copper mesh
CNT	carbon nanotubes
CNT-SA	carbon nanotubes with stearyl alcohol
CPCM	compounding phase change material
DOD	depths of discharge
DSC	differential scanning calorimetry
EG	expanded graphite
EP	expanded perlite
EV	electric vehicle
EVA	ethylene-vinyl acetate
GNP	graphene nano-platelet
HEV	hybrid electric vehicles
HP	heat pipe
HRR	heat release rate
LGPCM	liquid-gas phase change material
LIB	lithium-ion battery
MCH	magnesium chloride hexahydrate (MgCl_2_·6H_2_O)
MCMB	meso carbon microbeads
MPCM	microencapsulated phase change material
MWCNT	multi-walled carbon nanotube
NDG	nitrogen-doped graphene
NP	nickel particles
NREL	National Renewable Energy Laboratory
PA	paraffin
PCC	phase change composite
PCM	phase change material
PDA	polydopamine
PMMA	polymethyl methacrylate
SCF	short carbon fibers
SEI	solid electrolyte interface
SEM	scanning electron microscopy
SGPCM	solid-gas phase change material
SLPCM	solid-liquid phase change material
SSPCM	solid-solid phase change material
SWCNT	single-walled carbon nanotube
TCE	thermal conductivity enhancer
TCU	thermal control unit
TEM	transmission electron microscope
TES	thermal energy storage
TG	thermogravimetric
TR	thermal runaway
xGnP	exfoliated graphene nano-platelet
